# Pilot study of a brief provider and EMR-based intervention for overweight teens with asthma

**DOI:** 10.1186/s40814-021-00848-6

**Published:** 2021-08-30

**Authors:** Christine L. M. Joseph, Gwen L. Alexander, Mei Lu, Stacy L. Leatherwood, Rachel Kado, Heather Olden, Christina Melkonian, Cheryl A. Miree, Christine Cole Johnson

**Affiliations:** 1 Department of Public Health Sciences, Henry Ford Health System, 1 Ford Place, 3E, Detroit, MI 48202 USA; 2 Department of Pediatrics, Henry Ford Health System, Detroit, MI 48202 USA; 3 Division of Allergy and Immunology, Henry Ford Health System, Detroit, MI 48202 USA

## Abstract

**Introduction:**

Asthma-related morbidity is increased in overweight patients, yet providers are given little guidance on how to discuss weight and asthma management with overweight teens.

**Objective:**

We piloted an electronic medical record (EMR)-based tailored discussion guide (TDG) and a brief provider training, to address weight management in overweight teens with asthma. The primary outcome was intervention impact on patient-reported asthma outcomes (e.g., asthma control and morbidity). Secondary outcomes included change in BMI, patient-centeredness, and change in healthy behaviors.

**Methods:**

Teens aged 13–18 years with persistent asthma and a body mass index ≥ 85th percentile for their age and sex were eligible. Parents of eligible teens were contacted before an upcoming appointment to allow teen enrollment during the clinic visit. Providers reviewed Motivational Interviewing (MI) concepts and were trained in the TDG for support of conversations around weight and asthma management. Measures included asthma outcomes retrieved from the EMR at 6- and 12-month post-baseline, teen impressions of patient-provider communication at 6-week post-enrollment, and teen report of healthy behaviors at 6- and 12-month post-baseline.

**Results:**

Of 44 teens enrolled (77% African-American, 63% female), mean BMI for intervention (*n*=25) and control groups (*n*=19) at baseline were similar. Thirty participants (68%) completed a 6-week questionnaire. Compared to controls, at 6 months, intervention teens reported fewer days of limited activity and “uncontrolled asthma,” but at 12 months, only restricted activity remained lower, and BMI was not reduced. Intervention teens reported clinic visits that were more patient-centered than controls, including discussion of asthma treatment options with provider, feeling ready to follow an asthma treatment routine, and receiving helpful tips about reaching a healthy weight. The healthy behavior “dinner with family” showed improvement for intervention teens at 6 and 12 months. The feasibility study also revealed a need to improve recruitment strategies and to streamline intervention delivery.

**Conclusion:**

Modest improvements in patient-reported asthma outcomes and health behaviors were observed. There was strong evidence that the TDG supports provider discussion of weight and asthma to create a more patient-centered conversation from the perspective of participating teens. Challenges to recruitment and clinic adaptation must be addressed before advancing to a full-scale trial.

**Trial registration:**

NCT02575326 Teen Asthma Control Encouraging a Healthier Lifestyle, www.cllinicaltrials.gov

## Key messages regarding feasibility

- What uncertainties about feasibility existed prior to this study?
  Currently unknown is whether a tailored discussion guide embedded in the electronic medical record can facilitate provider discussion of weight and asthma with adolescent patients, promoting improved asthma outcomes and patient perception of engagement.
- What are the key findings on feasibility?
  Results show modest benefit for indicators of asthma control and patient-centeredness, but less so for healthy behaviors. In addition, benchmarks for recruitment goals were not met, and refusals were higher than expected. While no benchmarks for implementation were specified a priori, informal conversation with providers eluded to challenges in intervention adaptability.
- What are the implications of the findings on the design of the main study?
  In advance of a larger trial, this study revealed the need for improvements in recruitment approach and for strategies to streamline intervention design and delivery from both the patient and provider perspective.

## Introduction

Obesity and asthma independently represent public health challenges for urban communities [1]. Childhood asthma that has persisted into adolescence is likely to persist into adulthood [2]. For many Americans, adolescence is also a period in which they begin to experience weight gain, particularly when approaching the final stages of puberty [3]. Management of asthma may be more of a challenge in the overweight adolescent compared to normal weight youth [1]. Studies show higher asthma-related morbidity in this group, usually demonstrated through higher health care utilization for exacerbations [4]. Some reports have shown that overweight/obesity can adversely impact the effectiveness of asthma therapy [5, 6]. These and other findings suggest that interventions designed to achieve asthma control in overweight adolescents are necessary.

Given the sensitive nature of body image and weight management among adolescents, a patient-centered approach to opening the discussion around asthma control through weight management is needed [7]. Patient-centered care (or patient-focused care) involves a partnership between patient and provider that includes shared-decision making, productive communication, and health promotion [8]. In its 2001 report, “Crossing the Quality Chasm: A New Health System for the 21^st^ Century,” the Institute of Medicine’s action plan for achieving quality care across the nation included patient-centeredness among the 6 key components to reaching this goal [9]. This concept is also included in the Patient Protection and Affordable Care Act [10]. The objective of patient-centered care is to find treatment goals that satisfy the needs and expectations of the patient as well as the provider [11].

Patients often need assistance in achieving a healthy weight, yet little guidance is given to providers caring for obese adolescents with asthma [7]. Health care professionals play an important role in helping patients understand the role of weight loss in asthma control. Patients who receive this advice from their provider are more likely to report weight loss attempts [12]. Obese patients may require a conversation with greater sensitivity surrounding weight loss advice and the explanation of how weight status is connected to adverse outcomes for asthma [4]. In an earlier publication, we reported that adolescents feel it is appropriate for providers to initiate a weight management conversation in relation to asthma, but preferred conversation starters that recognized the challenges and included family participation in discussions around nutrition and physical activity [7]. Certain phrases such as “carrying around too much weight” were not popular with participants. Results suggested that providers may benefit from guidance on how best to initiate and fortify the conversation.

In this paper, we report the results of a pilot study for which the overall goal was to evaluate the feasibility of a brief, provider-based intervention combining provider training/review of motivational interviewing (MI) tenets and a tailored discussion guide (TDG) comprising discussion prompts embedded in the electronic medical record (EMR). To determine feasibility, we assessed intervention impact by randomizing participants to an intervention group (TDG) or a comparison group (standard care) and comparing selected patient-reported asthma indicators of control and morbidity collected from the EMR and through follow-up surveys as our primary outcome. Additional secondary outcomes included patient-reported perception of the patient-centeredness of a clinical encounter serving as the index visit for delivery of a provider-based intervention, and participant self-report of changes in healthy behaviors. We also evaluated recruitment, expecting less than ≤ 20% refusal to participate, and the ability to successfully reach targeted enrollment.

## Methods

All aspects of this research were approved by the Henry Ford Health System Institutional Review Board. This is a cluster randomized pilot study in which the provider was the unit of randomization.

### Patient identification and recruitment

We used our electronic medical record (EMR) to identify youth aged 13–18 years with persistent asthma and a BMI ≥ 85th percentile by applying criteria from the Healthcare Effectiveness Data and Information Set (HEDIS) denominator for the “Use of Appropriate Medications for People with Asthma” measure [13]. Information for these patients was linked to the centralized appointment scheduling system to generate a listing of eligible patients with upcoming appointments using a 30-day window. The listing was refreshed weekly to include patients that had recently become eligible or were new to the health care system and may have been candidates for enrollment. Research staff introduced the study to potential participants in the clinic waiting area and obtained parental informed consent and teen assent. The teen was then asked to complete the baseline questionnaire. After the index visit, teens were emailed a link to follow-up surveys asking for feedback on the index visit at 6 weeks and to obtain study outcomes at 12 months. The recruitment/follow-up period was August 2015–February 2018.

### Randomization

Pediatricians seeing adolescents in 8 clinics and consenting to the randomization were randomized to an intervention or standard care (control) group. After completing the consent and assent process described above, patients with scheduled appointments with intervention providers were analyzed as the intervention group, and teens visiting the control providers were assigned the control group.

### Intervention

The intervention consisted of provider training or review in the principles of motivational interviewing (MI) [14]. A training module was developed under the guidance of an expert in health behavior who was certified in MI (G.A.). Providers consenting to participate in the study attended a scheduled training session at their clinic which lasted about 1 h. The content of the training focused on known tenets of MI, including reflections, asking permission, expressing empathy, rolling with resistance, and supporting self-efficacy. The intent of the training was to help providers by reinforcing and demonstrating how to adopt a more patient-centered approach to incorporating attention to achieve healthy weight into discussions about asthma management. Aspects might include listening to and reflecting the patient’s take on key issues, identifying and motivating with patient preferences in mind, encouraging patients to create a first step and take personal responsibility, and uncovering and facilitating reflection on patient *conflict* between motivation and resistance.

The training session also included guidance on how to access and use the EMR-based TDG during an office visit. Participants were asked to complete a brief self-administered online questionnaire in the waiting room during the visit at which the teen was recruited (index visit) and before the patient was taken to an exam room to see the doctor. This questionnaire included the Asthma Control Test (ACT), the Asthma Therapy Assessment Questionnaire (ATAQ), questions on awareness of links between weight and asthma, and questions on eating habits and activities and feelings about managing overweight. The ACT scores were entered separately into the patient’s EMR by research staff, while the other teen responses were linked electronically to the patient’s EMR. Using Epic smart-phrases, weight and asthma conversation prompts for intervention providers appeared in the EMR and were tailored to the adolescent participant’s questionnaire responses. The provider was asked to begin the discussion with asthma management after which he/she would ask the patient’s permission to launch the discussion about asthma and overweight. Conversation prompts began with the ACT score and results from the ATAQ to determine asthma control and satisfaction with current asthma treatment regimen. With this information, the provider could apply the principles described above, including expressing empathy as appropriate, discovering patient values, and obtaining the patient perception of the importance of asthma control and a healthy weight. The decision guide prompted the visit to end with the patient selection of a weight-related behavior to change, an assessment of the patient’s confidence that he/she would be able to make that change, review of agreed-upon short-term goals, and a scheduling of the next visit. Providers who were randomized to standard care only received information on the consent and assent process for patient enrollment, incentives, and follow-up. BMI at 6 and 12 months was collected from the EMR. Patients were provided $40 for the index clinic visit (enrollment) and $20 for each follow-up completed.

### Statistical analysis

To describe our results, clustered analyses were performed, with the provider (representing a cluster of patients) as the unit of analysis. A propensity score was used to address imbalances in participant characteristics at baseline and in the analysis of study outcomes. Clustered regression with a logit link function was used for binary outcome, and generalized regression was used for analysis of follow-up survey score outcome. Ninety-five percent confidence intervals were calculated for the estimated mean difference.

## Results

**Fig. 1:**
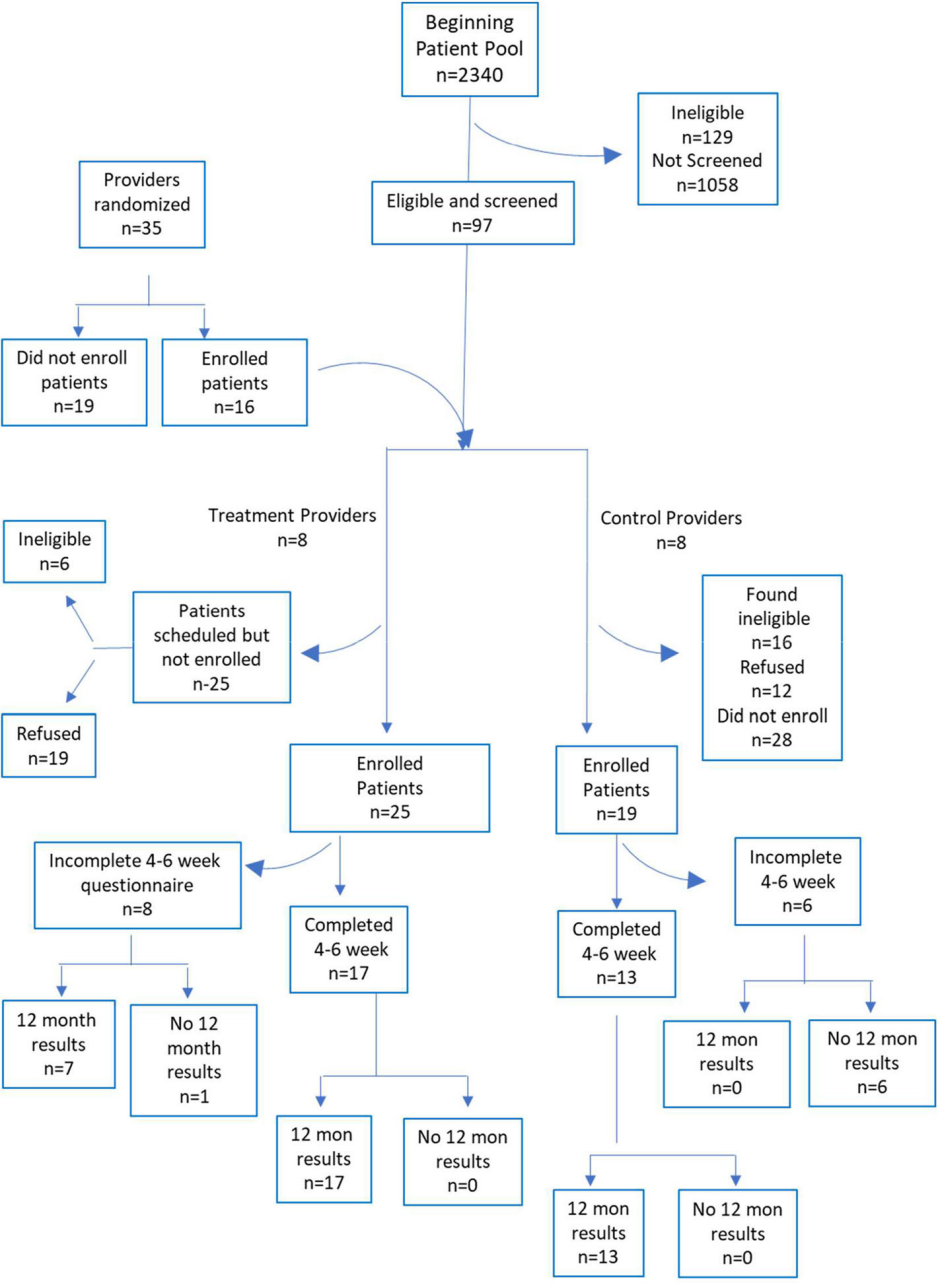
Flow diagram of study and analytic sample

A total of 16 providers, 8 in the intervention group and 8 in control group, were enrolled. Of the 1153 appointments identified for potentially eligible patients, a total of 97 teens were approached, of whom 44 (45.4%) were eligible and consented to participate (Fig. 1). Of the 97 adolescents approached, 22.7% were ineligible, of which the majority (68.2%) did not meet BMI criteria for enrollment at the time of the visit. Six of this group were between BMI percentile of >79 <80, and another six were between BMI percentile of >69 <79. A total of 31 patients refused (32%). Of the participants consenting to enroll, 77% were African-American, 63% were female, and mean age =16.21 (sd=1.37). At baseline, treatment and control participants did not differ by mean age, race, mean ACT score, Medicaid enrollment, or a Detroit versus suburban zip code. Mean BMI for intervention and control teens was 33.8 (94.5th percentile) and 31.64 (95.4th percentile), respectively. There were more females in the treatment group (76%) than the control group (44%), and so all analyses were adjusted for sex. Consenting teens had scheduled appointments with enrolled providers randomized to the intervention (*n*=25) or control group (*n*=19). Thirty participants (*n*=17 [56.7%] intervention and *n*=13 [43.3%] control) completed the 6-week questionnaire (Fig. 1).

Table 1 shows results for indicators of control and morbidity at 6 and 12 months. Overall, at 6 months, youth in the intervention group had better indicators of asthma control as indicated by the mean difference (control estimated mean − treatment estimated mean). However, only the mean difference (MD) calculated for mean days of limited activity and perception of asthma control had corresponding 95% CI that did not contain 0, MD=1.08 (0.08, 2.09) and 0.41 (0.21, 0.62) for limited activity and asthma control, respectively. There were no 6-month comparisons for morbidity and healthcare utilization in which the 95% CI did not contain 0, although a trend toward lower asthma control and morbidity was observed. For morbidity, the treatment group had fewer asthma episodes, MD=1.16 (0.47, 1.85).

**Table 1:** Six- and 12-month follow-up outcomes for feasibility study of provider tailored discussion guide: asthma control and management

	6-month follow-up						12-month follow-up					
Outcome	Control		Treatment	Estimated mean difference (95%CI)			Control		Treatment		Estimated mean difference (95%CI)	
Indicators of control*, mean (SE)												
Wheeze/30 days	1.56	(0.21)	0.93	(0.29)	0.63	(−0.15, 1.42)	1.92	(0.69)	1.08	(0.19)	0.84	(−0.69, 2.37)
Used rescue inhaler	0.71	(0.25)	0.56	(0.31)	0.14	(−0.71, 1.00)	1.49	(0.45)	0.79	(0.14)	070	(−0.30, 1.71)
Nights awakened	0.91	(0.36)	0.25	(0.07)	0.66	(−0.13, 1.45)	1.49	(0.43)	0.67	(0.11)	0.79	(−0.16, 1.74)
Limited activity	2.21	(0.46)	1.12	(0.08)	1.08	(0.08, 2.09)	1.79	(0.24)	0.87	(0.14)	0.91	(0.31, 1.51)
Missed school	0.20	(0.15)	0.12	(0.10)	0.08	(−0.32, 0.48)	0.86	(0.40)	0.29	(0.10)	0.57	(−0.32, 1.45)
Days perceived asthma uncontrolled	1.04	(0.03)	0.62	(0.09)	0.41	(0.21, 0.62)	1.31	(0.37)	0.67	(0.10)	0.64	(−0.17, 1.46)
**Morbidity, mean (SE)**												
Symptom-free/14 days	9.96	(1.57)	17.75	(7.45)	−7.79	(−24.24, 8.6)	6.8	(2.28)	10.21	(0.71)	−3.39	(−8.51, 1.73)
Asthma episodes/6 months	2.77	(1.55)	1.19	(0.44)	1.59	(−1.89, 5.07)	1.54	(0.30)	0.37	(0.12)	1.16	(0.47, 1.85)
ED visit	0.30	(0.26)	0.19	(0.10)	0.12	(−0.48, 0.72)	0.34	(0.27)	0.29	(0.06)	−0.15	(−0.49, 0.19)
Hospital admissions	0		0			N/A	0.48	(0.27)	0.32	(0.09)	0.16	(−0.45, 0.77)

We observed that 15 out of 17 items measuring patient-centeredness of the encounter had MD indicating a more patient-centered visit for treatment versus control participants, i.e., a negative MD indicating higher ratings (more patient-centered) for intervention teens compared to controls (Table 2). Compared to controls, intervention teens were more likely to report discussing asthma treatment options with the provider, MD=−1.44 (−2.33, −0.55), and reported feeling ready to follow an asthma treatment routine, MD=−1.08 (−1.54, −0.63). Intervention teens were also more likely to report that the provider offered helpful tips about reaching a healthy weight, MD=−0.55 (−1.08, −0.02).

**Table 2:** Results of participant assessment of patient-centered visit on asthma and weight management at 4–6 weeks after the study initiation

		8 clusters/13 teens	8 clusters/17 teens	Estimated mean difference^a^	(95% CI)
Statement		Control	Treatment		
1.	Asked my provider questions.	2.00	3.44	−1.44	(−2.33, −0.55)
2.	Ready to follow asthma treatment.	3.23	4.31	−1.08	(−1.54, −0.63)
3.	Felt encouraged to express my thoughts.	3.98	3.94	0.05	(−0.67, 0.76)
4.	Provider listened carefully.	4.05	4.44	−0.39	(−1.48, 0.70)
5.	Provider discussed asthma treatment options.	3.77	4.32	−0.55	(−1.08, −0.02)
6.	Provider checked to see if plan works	2.73	4.00	−1.27	(−2.94, 0.40)
7.	Provider encouraged me to ask questions.	2.94	4.00	−1.06	(−2.98, 0.85)
8.	Provider responded to my questions.	4.36	4.19	0.17	(−0.37, 0.71)
9.	Provider involved me in decisions.	3.07	4.25	−1.18	(−2.41, 0.04)
10.	Provider discussed next steps.	3.77	4.13	−0.36	(−0.87, 0.16)
11.	Provider spent right amount of time.	3.96	4.19	−0.22	(−0.62, 0.19)
12.	Provider checked to make sure I understood.	3.97	4.44	−0.47	(−0.77, −0.17)
13.	Past asthma problems taken into account during this visit.	2.93	4.44	−1.51	(−2.47, −0.55)
14.	Provider gave me help to maintain healthy body weight.	2.22	3.94	−1.72	(−3.35, −0.09)
15.	Provider talked to me about improving my health.	2.97	4.38	−1.41	(−3.29, 0.48)
16.	Provider helped me change my habits.	2.78	4.19	−1.41	(−3.21, 0.40)
17.	Provider asked if my current weight makes it hard to do things.	2.58	4.00	−1.42	(−2.93, 0.09)

In the 6-month comparison of healthy behaviors (Table 3), intervention teens were less likely to spend time in physical activity, MD=1.63 (0.07, 3.19), but were more likely to have dinner with the family, MD=−1.76 (−3.50, −0.01), which was also observed at 12 months, MD= −1.87 (−3.31, −0.42). BMI increased slightly for teens in the intervention group and in the control group, but 95%CI for MD=−0.14 contained 0.

**Table 3:** Six- and 12-month follow-up outcomes for feasibility study of provider tailored discussion guide: healthy behaviors

	6-month follow-up						12-month follow-up					
**Outcome**	**Control**		**Treatment**		**Estimated mean difference (95%CI)**		**Control**	**Treatment**		**Estimated mean difference (95%CI)**		
**Healthy habits, mean (SE)**												
Servings fruits and veg/day	2.94	(0.48)	2.0	(0.22)	0.94	(−0.21, 2.10)	2.2	(0.28)	1.8	(0.15)	0.38	(−0.30, 1.1)
TV time	3.54	(1.12)	2.94	(0.26)	0.61	(−1.89, 3.10)	3.46	(1.04)	2.79	(0.23)	0.67	(−1.6, 2.96)
Activity time	4.01	(0.69)	2.37	(0.21)	1.63	(0.07,3.19)	3.51	(0.50)	2.75	(0.33)	0.76	(−0.52, 2.04)
Sugary drinks	2.32	(0.17)	2.31	(0.27)	0.004	(−0.68, 0.69)	2.07	(0.46)	2.00	(0.20)	0.08	(1.00, 1.15)
Hours of sleep	5.59	(0.69)	6.87	(0.31)	−1.29	(−2.93, 0.36)	6.69	(0.27)	6.87	(0.28)	−0.19	(−1.02, 0.65)
Eat breakfast	1.78	(0.90)	2.0	(0.29)	−0.23	(−2.27, 1.82)	1.60	(0.43)	2.29	(0.42)	−0.69	(−2.0, 0.61)
Dinner with family	1.24	(0.65)	3.0	(0.48)	−1.76	(−3.50, −0.01)	1.09	(0.63)	2.96	(0.25)	−1.87	(−3.31, −0.42)
BMI *z* score at 12 months							1.74	(0.31)	1.88	(0.13)	−0.14	(−0.87, 0.59)

**Fig. 2:**
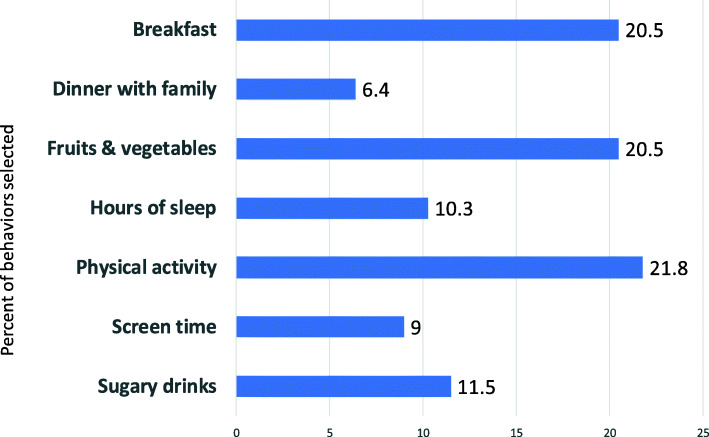
Healthy habits selected by participants

Figure 2 shows the distribution of health behaviors selected by persons in the treatment group. Participants could select more than one behavior. Physical activity made up 21.8% of all health behaviors selected, followed by an increase in servings of fruits and vegetables (20.5%) and eating breakfast (20.5%).

We collected comments from six participating physicians who agreed to give brief feedback. Four of the six physicians providing feedback said that the guide facilitated the discussion about weight management and asthma; however, all four agreed that the TDG did lengthen the appointment. Three of the six providing feedback said they would continue to use the TDG; 1 was neutral and the other would not continue to use the guide.

## Discussion

Providers and patient reach “common ground” when they share treatment goals, agree on a management approach, and understand each other’s reasoning for the choices made [8, 11]. We developed an intervention targeting adolescents with asthma and a BMI > 85th percentile. The intervention involved a combination of provider training/review of MI principles and use of a tailored discussion guide (TDG) with conversation prompts embedded in the EMR to be used during a single clinical encounter. To assess the feasibility of evaluating this intervention in a larger trial, we assessed intervention impact on indicators of asthma control and morbidity, as well as participant assessment of patient-centeredness of the intervention, and intervention impact on self-report of healthy behaviors. Results in the expected direction were observed for items in all three of these areas. Our pilot also revealed challenges in areas not necessarily targeted in this feasibility study, such as recruitment and implementation issues, that are certainly applicable to conducting a larger trial.

We did observe significantly fewer asthma episodes in the treatment group, but after this single doctor-patient interaction, we did not see evidence of significant changes in BMI. Patient-centeredness was impacted according to our results; however, in this small pilot, intervention teens showed improvement only in some lifestyle behaviors, specifically eating dinner with family and physical activity. Eating dinner with family is believed to encourage good eating and to reduce stress [15, 16]. This was one of the behaviors that was assessed of all participants in the lifestyle behavior survey prior to their doctor visit, with hopes of generating reflection which might prompt behavior change and possible improved eating habits. However, fewer than 10% (*n*=2) selected this behavior. Increasing physical activity was selected by approximately 22% of intervention teens. At 12 months, report of physical activity was in the hypothesized direction for the treatment group, but we can only speculate about a decrease in physical activity among the controls. We note that the standard error for physical activity at 6 months for the controls is three times that of the treatment group, highlighting the need for a larger sample size.

Successful interventions in the literature with similar goals have been lengthier and more involved. A Dutch study of 87 children 6–16 years of age who were overweight/obese and either had asthma or were at high risk of developing asthma also reported no significant weight loss differences by group assignment; however, asthma indices improved in the intervention group compared to the controls [17, 18]. The 18-month intervention included sessions on physical activity, parental involvement, and individual counseling that included dietary advice and behavioral therapy [17, 18]. A Brazilian study of obese adolescents (*n*=76, 26 (34.2%) of whom had asthma) with a year-long intervention demonstrated reductions in asthma severity and beneficial changes in inflammatory biomarkers [19]. This study also included psychological and nutritional counseling weekly, along with exercise sessions 3 times per week [19]. Shorter-term interventions have had some success. For example, Jensen et al., in Australia, used diet-focused strategies in a 10-week multi-visit program designed to reduce weight in obese children 8–17 years with asthma. Short-term weight loss was observed along with lung function improvements and asthma control, although indicators of inflammation did not change [20].

Almost 23% of potential participants were ineligible to enroll, mostly due to BMI inclusion criteria, and this should be noted in terms of reaching targeted enrollment for a subsequent trial. Recruitment was most challenged by willingness of parents and their teens to enroll and attrition. Targeted enrollment goals were not met, and slightly less than 70% of those enrolled completed the follow-up survey. Possible improvements include better messaging and strategies to encourage patients and caregivers to participate, along with immediate research incentives (i.e., intervention participants must wait to experience health improvements due to motivated weight loss). Interventions reported in the literature with similar objectives included behavioral therapy and dietary information, which our brief intervention did not include, although referrals were possible at the provider’s discretion. This study’s small sample size, high refusal rate, and considerable attrition could have resulted in a biased sample of teens who differ from non-participants in terms of motivation to participate and study compliance, which could impact the validity of a larger trial. Bias could also be introduced due to control providers being aware of the link between asthma and obesity because of the study and feeling led to emphasize weight management in discussions about asthma with control study patients. If this were the case, we might expect our results to be biased toward the null, making it more difficult to observe differences. At the time of writing this manuscript, we were unable to find similar US provider-based interventions targeting weight loss among adolescent patients with asthma in the US. Provider feedback suggested that the short “well visit” appointment time frame of the conversation and use of the TDG did not allow time of in-depth discussion on behavioral changes and reported that the TDG increased the length of the appointment, which would be detrimental to its adaptation. Providers suggested delivering the intervention content in two visits instead of one to better fit the primary care setting, but we found that participants are less likely to return for the second visit to discuss weight management, despite an incentive to defray any associated costs (e.g., parking or childcare).

In summary, the literature suggests that 12% of adolescents with asthma are also overweight [21], and this percentage can increase 3-fold among African-American youth [22]. Developing and evaluating provider-based strategies to encourage a healthy weight in overweight patients with asthma is essential to managing asthma in this population. Asthma patients who report better communication and partnership with a provider are more adherent to treatment regimens and have better clinical outcomes [3, 23]. We proposed that a brief intervention in the form of a tailored discussion guide that quickly identified key patient characteristics and preferences may improve patient-provider communication when combined with provider review of MI concepts. We felt that this focus on patient-centered outcomes would result in patients intrinsically motivated to adhere to recommended regimens (Fig. 2). This pilot revealed evidence of intervention impact on asthma outcomes, patient-centeredness, and selected behaviors. Modifications to this intervention in terms of study design and recruitment strategies and parameters that would impact provider adaptation of the TDG are needed to fully assess its impact by conducting a larger trial in this population.

## Data Availability

The datasets used and/or analyzed during the current study are available from the corresponding author on reasonable request.

## Abbreviations

ACT: Asthma Control Test
ATAQ: Asthma Therapy Assessment Questionnaire
BMI: Body mass index
EMR: Electronic medical record
MI: Motivational interviewing
TDG: Tailored discussion guide
